# Simple-to-use nomogram for evaluating the incident risk of moderate-to-severe LEAD in adults with type 2 diabetes: A cross-sectional study in a Chinese population

**DOI:** 10.1038/s41598-019-55101-1

**Published:** 2020-02-21

**Authors:** Xin Zhao, Xiaomei Zhang, Xingwu Ran, Zhangrong Xu, Linong Ji

**Affiliations:** 1 Department of Endocrinology, Peking University International Hospital, Beijing, 100001 China; 2 Department of Endocrinology, West China Hospital, Sichuan University, Sichuan, 610041 China; 3 Diabetes Center, Department of Endocrinology, The 306th Hospital of PLA, Beijing, 100001 China; 4 Department of Endocrinology, Peking University People’s Hospital, Beijing, 100001 China

**Keywords:** Type 2 diabetes, Type 2 diabetes

## Abstract

This study aimed to analyze the clinical characteristics of lower extremity atherosclerotic disease (LEAD) in Chinese adult type 2 diabetes (T2D) patients, and also explored the risk factors for LEAD and developed simple-to-use nomograms for LEAD and lesion degree in these patients. We retrospectively studied 4422 patients (male = 2084; female = 2338) with T2D who were ≥50. Based on lower extremity arterial ultrasound findings, we divided the patients into three groups: normal, mild, and moderate-to-severe group. The factors related to LEAD in patients with T2D were analyzed by logistic regression analysis. The risk factors for moderate-to-severe LEAD included: high HbA1c (OR = 1.07 95% CI 1.02–1.13), diabetic peripheral neuropathy (OR = 1.93 95% CI 1.57–2.37), and diabetic retinopathy (OR = 1.26 95%CI 1.01–1.57). The overall areas under the receiver operating characteristic curves for the nomograms for predicting the risks of LEAD and moderate-to-severe LEAD in adult T2D patients were 0.793 (95%CI 0.720, 0.824) and 0.736 (95%CI 0.678, 0.795), respectively. The developed nomograms are simple to use and enable preliminary visual prediction of the risk and degree of LEAD in Chinese T2D patients over 50 years. The nomograms are accurate to a certain degree and provide a clinical basis for predicting the occurrence and progression of LEAD.

## Introduction

The International Diabetes Federation (IDF) reported the Global Diabetes Map in 2017 with statistics for 425 million adults suffering from diabetes worldwide (with a prevalence rate of 8.8%). It also predicted that this number will increase to 629 million by 2045^1^. The 2013 representative cross-sectional survey data for mainland China showed that the prevalence rate of adult diabetes in China was as high as 10.9%^2^. Lower extremity arterial stenosis or occlusion causes lower extremity atherosclerotic disease (LEAD). Peripheral artery disease (PAD) manifests mainly as LEAD in patients with T2D. LEAD is a serious comorbidity of diabetes and commonly leads to disability and amputation in T2D patients^3^. However, the clinical symptoms are often not obvious, and thus, the diagnosis is complicated. Even after diagnosis, treatment of LEAD requires hospitalization, and thus, is associated with high healthcare costs^4^.

Previous research studies have shown that LEAD and gender, age, body mass index (BMI), cigarette smoking, alcohol consumption, hypertension, HbA1c level, diabetic nephropathy, retinopathy, and other factors are closely related. However, the results have not been consistent among different studies^5–7^. Traditional factor analyses have revealed the risk factors for LEAD in specific populations but could not visually display the risk of LEAD in diabetes individually. Therefore, our study aimed to develop a simple-to-use nomogram for the prediction of the risk as well as the degree of LEAD in adult T2D patients. Such a tool will help provide early evidence for a high risk of LEAD among T2D patients with T2D for who effective preventive or therapeutic measures should be applied to prevent further dysfunction. Also, a clear understanding of the key risk factors for LEAD will provide a basis for prevention and delay of serious complications.

## Results

### Baseline characteristics and comparison among groups

The normal, mild LEAD, and moderate-to-severe LEAD groups consisted of 1779, 1242, and 1401 patients, respectively. The mean age of patients with moderate-to-severe LEAD was higher than those of other groups (p < 0.05), and the smoking rates of the mild and moderate-to-severe groups were higher than that of the normal group, with a statistically significant difference among the three groups (p < 0.05). The proportions of males in the mild and moderate-to-severe groups were higher compared with that in the normal group (p < 0.05). Patients with mild and moderate-to-severe LEAD had a longer diabetic duration and higher level of SBP, p < 0.05) compared to T2D patients without LEAD. The proportion of patients with CHD, CBD, neuropathy, retinopathy, and nephropathy were higher in the mild and moderate-to-severe LEAD groups than in the normal group (p < 0.05). However, the proportions of drinking, T2D history among first-degree relatives, CHD, and CBD (p > 0.05) showed no significant differences among the groups. The TC, TG, LDL-C, and HbA1c levels as well as the eGFR differed significantly among the three groups (all p < 0.05), whereas the other indices (p > 0.05) showed no significant differences (Table 1).

**Table 1:** Comparison of general baseline characteristics among the three patient groups.

Index	Normal group (n = 1779)	Mild Group (n = 1242)	Moderate-to-severe Group (n = 1401)	F (χ^2^)	p
Age (years)	63.39 ± 8.16	63.84 ± 8,78	64.23 ± 9.06	3.76	<0.05
Sex				71.94*	<0.05
Male	730(41.03%)	569(45.81%)	785(56.03%)		
Female	1049(58.97%)	673(54.19%)	616(43.97%)		
Smoking (%)	297(16.69%)	272(21.90%)	379(27.05%)	50.14*	<0.05
Drinking (%)	266(14.96%)	191(15.48%)	222(15.88%)	0.52*	0.77
Duration (years)	8.98 ± 7.91	9.95 ± 7.44	10.15 ± 7.16	11.09	<0.05
BMI (kg/m^2^)	24.52 ± 2.83	24.45 ± 3.46	24.64 ± 3.57	1.10	0.33
Family history (%)	241(15.02%)	170(14.06%)	192(14.04%)	0.75*	0.69
SBP (mmHg)	135.43 ± 18.95	136.98 ± 18.90	137.12 ± 18.94	3,70	<0.05
DBP (mmHg)	79.23 ± 11.28	79.46 ± 11.63	80.09 ± 11.40	2.19	0.11
TC (mmol/L)	4.35 ± 1.32	5.01 ± 1.23	5.01 ± 1.03	137.75	<0.05
TG (mmol/L)	1.70 ± 1.44	1.61 ± 1.08	1.89 ± 1.27	34.59	<0.05
LDL-C (mmol/L)	2.55 ± 0.97	2.91 ± 0.99	2.91 ± 1.06	58.63	<0.05
HDL-C (mmol/L)	1.06 ± 0.46	1.13 ± 0.28	1.18 ± 0.34	3.02	0.12
eGFR (mL/min/1.73 m^2^)	101.65 ± 30.80	105.01 ± 29.12	105.42 ± 29.19	5.50	<0.05
UA (mmol/L)	321 ± 108.91	308.13 ± 91.88	329.13 ± 97.00	2.34	0.24
BUN (mmol/L)	7.63 ± 1.02	7.72 ± 0.98	7.46 ± 1.65	1.23	0.89
HbA1c (%)	8.47 ± 2.27	8.48 ± 2.06	8.74 ± 2.22	6.90	<0.05
CHD (%)	193(10.85%)	163(13.12%)	199(144.20%)	8.56	<0.05
CBD (%)	241(13.55%)	209(16.83%)	244(17.42%)	10.55*	<0.05
Complications					
Nephropathy (%)	224(12.59%)	304(24.48%)	409(29.19%)	140.54*	<0.05
Neuropathy (%)	530(29.79%)	616(49.60%)	953(68.02%)	462.54*	<0.05
Retinopathy (%)	242(13.60%)	349(28.10%)	509(36.33%)	226.26*	<0.05

BMI, body mass index; SBP, systolic blood pressure; DBP, diastolic blood pressure, HbA1c, glycosylated hemoglobin; TC, total cholesterol; TG, triglyceride; LDL-C, low-density lipoprotein cholesterol; HDL-C, high-density lipoprotein cholesterol; UA, uric acid; BUN, blood urea nitrogen; eGFR, estimated glomerular filtration rate; CHD, coronary heart disease; CBD, cerebrovascular disease. *Indicates χ^2^ test result.

### Analysis of risk factors for LEAD and moderate-to-severe LEAD

Multicollinearity analysis was used for univariate logistic regression analysis of patients’ baseline characteristics (age, gender, diabetes duration, smoking, HbA1c, CHD, CBD, diabetic nephropathy, diabetic retinopathy, and diabetic neuropathy). No collinearity was identified among the factors, and the variance inflation factor (VIF) was <5 for all factors.

Multivariate logistic regression analysis was performed with LEAD as a dependent variable and baseline characteristics (age, gender, diabetic duration, smoking, HbA1c, CHD, CBD, diabetic nephropathy, diabetic retinopathy, and diabetic neuropathy) as independent variables. The risk factors for LEAD included: male gender (OR = 1.87, 95CI% 1.57–2.29), advanced age (OR = 1.02 95CI% 1.01–1.03), longer diabetic duration (OR = 1.03 95CI% 1.02–1.03), smoking (OR = 1.12 95CI% 1.07–1.19), higher HbA1c level (OR = 1.02 95CI% 1.01–1.03), CHD (OR = 1.38 95CI% 1.07–1.79), diabetic nephropathy (OR = 1.50 95CI% 1.20–1.88), diabetic neuropathy (OR = 2.10 95CI% 1.76–2.51), and diabetic retinopathy (OR = 2.64 95CI% 2.11–2.90). These data indicate associations with the risk of LEAD in patients with T2D after adjustment for BMI, blood pressure (BP), lipid levels, and eGFR (Table 2).

**Table 2:** Multivariate unconditional logistic regression analysis for risk of LEAD.

Index		B	OR	95% CI	p
Sex	Model 1	0.38	1.40	(1.21, 1.62)	<0.05
	Model 2	0.63	1.87	(1.57, 2.24)	<0.05
Age	Model 1	0.24	1.05	(1.03, 1.08)	<0.05
	Model 2	0.02	1.02	(1.01, 1.03)	<0.05
Duration	Model 1	0.02	1.02	(1.01, 1.03)	<0.05
	Model 2	0.03	1.03	(1.02, 1.04)	<0.05
Smoking	Model 1	0.20	1.22	(1.02, 1.47)	<0.05
	Model 2	0.09	1.12	(1.07, 1.19)	<0.05
CHD	Model 1	0.23	1.31	(1.09, 1.54)	<0.05
	Model 2	0.32	1.38	(1.07, 1.79)	<0.05
CBD	Model 1	0.21	1.24	(1.03, 1.48)	<0.05
	Model 2	0.22	1.25	(0.98, 1.58)	0.07
HbA1c	Model 1	0.20	1.24	(1.03, 1.54)	<0.05
	Model 2	0.20	1.02	(1.01, 1.03)	<0.05
Nephropathy	Model 1	0.56	1.75	(1.47, 2.08)	<0.05
	Model 2	0.41	1.50	(1.20, 1.88)	<0.05
Neuropathy	Model 1	1.05	2.86	(2.50, 3.27)	<0.05
	Model 2	0.74	2.10	(1.76, 2.51)	<0.05
Retinopathy	Model 1	0.85	2.33	(1.97, 2.76)	<0.05
	Model 2	0.97	2.64	(2.11, 2.90)	<0.05

CHD, coronary heart disease; CBD, cerebrovascular disease. Model 1 is adjusted for BMI, and Model 2 is adjusted for BMI, BP, TC, TG, LDL-C, HDL-C, and eGFR.

Multivariate unconditional logistic regression analysis after adjustment for BMI, BP, lipid levels, and eGFR identified the following risk factors for moderate-to-severe LEAD: higher HbA1c level (OR = 1.07 95CI% 1.02–1.13), diabetic peripheral neuropathy (OR = 1.93 95CI% 1.57–2.37), and diabetic retinopathy (OR = 1.26 95CI% 1.01–1.57; Table 3).

**Table 3:** Multivariate unconditional logistic regression analysis for risk of moderate-to-severe LEAD.

Index		B	OR	95% CI	p
Sex	Model 1	0.35	1.42	(1.19, 1.71)	<0.05
	Model 2	0.05	1.03	(0.09, 1.42)	0.21
Age	Model 1	0.00	1.00	(0.99, 1.01)	0.40
	Model 2	0.01	1.01	(1.00, 1.02)	0.16
Duration	Model 1	0.00	1.00	(0.99, 1.01)	0.80
	Model 2	0.01	1.01	(0.99, 1.02)	0.32
Smoking	Model 1	−0.01	1.00	(0.80, 1.23)	0.93
	Model 2	0.11	1.12	(0.85, 1.47)	0.43
CHD	Model 1	0.04	1.04	(0.83, 1.32)	0.72
	Model 2	−0.17	0.85	(0.64, 1.13)	0.26
CBD	Model 1	0.03	1.03	(0.84, 1.28)	0.76
	Model 2	−0.02	0.98	(0.76, 1.26)	0.85
HbA1c	Model 1	0.08	1.09	(1.05, 1.13)	<0.05
	Model 2	0.07	1.07	(1.02, 1.13)	<0.05
Nephropathy	Model 1	0.05	1.05	(0.88, 1.26)	0.59
	Model 2	0.04	1.04	(0.83, 1.31)	0.73
Neuropathy	Model 1	0.74	2.10	(1.78, 2.47)	<0.05
	Model 2	0.66	1.93	(1.57, 2.37)	<0.05
Retinopathy	Model 1	0.28	1.32	(1.11, 1.57)	<0.05
	Model 2	0.23	1.26	(1.01, 1.57)	<0.05

CHD, coronary heart disease; CBD, cerebrovascular disease. Model 1 is adjusted for BMI, and Model 2 is adjusted for BMI, BP, TC, TG, LDL-C, HDL-C, and eGFR.

### Predictive accuracy of nomograms for LEAD and moderate-to-severe LEAD in T2D patients

**Figure 1:**
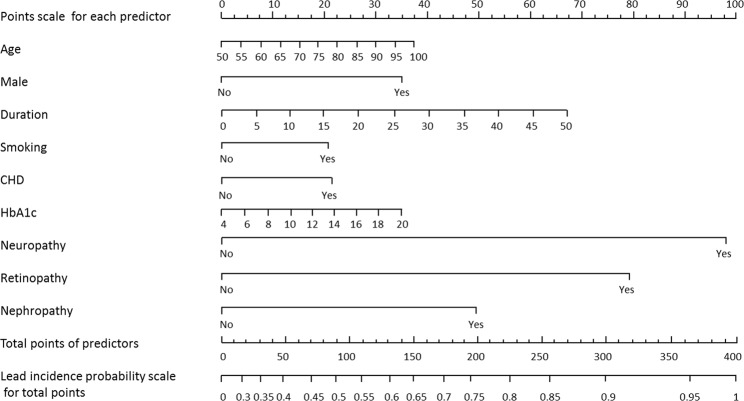
Nomogram for LEAD in patients with T2D. Instructions: Each individual’s LEAD risk was estimated by plotting on each variable axis. A verticle line was drawn from that value to the top points scale to determine the number of points that were assigned by that variable value. Then, the points from each variable value were summed. The sum on the total points scale was located and vertically projected onto the bottom axis, and then a personalized LEAD risk for T2D was obtained. CHD, chronic heart disease; LEAD, lower extremity atherosclerotic disease; T2D, type 2 diabetes.

**Figure 2:**
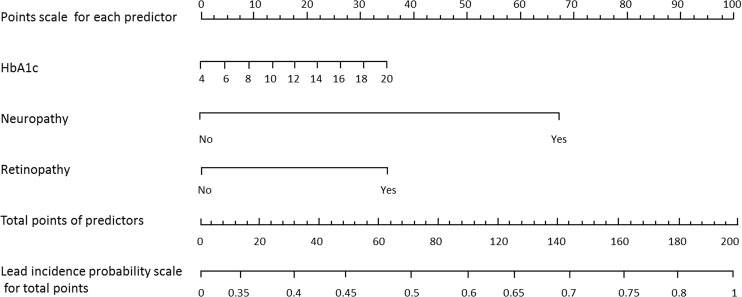
Nomogram for moderate-to-severe LEAD in patients with T2D LEAD. Instructions: To estimate the risk for moderate-to-severe LEAD among patients with T2D LEAD, each individual patient’s values were plotted on each variable axis. A verticle line was drawn from that value to the top points scale to determine the number of points that were assigned by that variable value. Then, the points from each variable value were summed. The sum on the total points scale was located and vertically projected onto the bottom axis, and then a personalized moderate-to-severe LEAD risk for T2D LEAD was obtained. LEAD, lower extremity atherosclerotic disease; T2D, type 2 diabetes.

**Figure 3:**
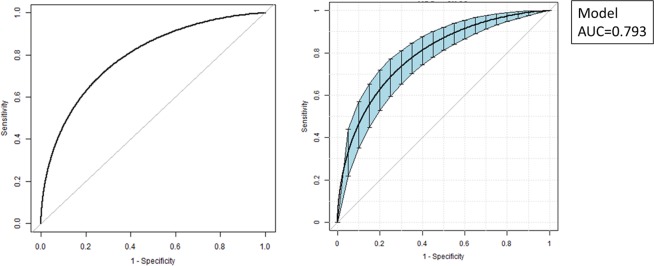
ROC curves for the accuracy of the LEAD nomogram in patients with T2D. The overall predictive accuracy of the nomogram for LEAD was 0.793 (95%CI 0.720, 0.824), and the sensitivity and specificity were 0.619 and 0.849, respectively. AUC, area under the curve; CI, confidence interval; LEAD, lower extremity atherosclerotic disease; ROC, receiver operating characteristic; T2D, type 2 diabetes.

**Figure 4:**
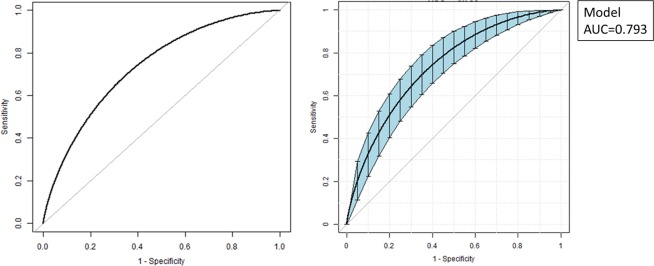
Receiver operating characteristic (ROC) curves for the accuracy of the moderate-to-severe LEAD nomogram in patients with T2D. The overall predictive accuracy of the nomogram for moderate-to-severe LEAD was 0.736 (95%CI 0.678, 0.795), and the sensitivity and specificity were 0.792 and 0.60, respectively. AUC, area under the curve; CI, confidence interval; LEAD, lower extremity atherosclerotic disease; ROC, receiver operating characteristic; T2D, type 2 diabetes.

Nomograms for evaluating the risk of LEAD (Fig. 1) and moderate-to-severe LEAD (Fig. 2) were developed for T2D patients based on risk factors identified by the multivariate logistic regression analysis. The overall predictive accuracy of the nomogram for LEAD was 0.793 (95%CI 0.720, 0.824), and the sensitivity and specificity were 0.619 and 0.849, respectively. The overall predictive accuracy of the nomogram for moderate-to-severe LEAD was 0.736 (95%CI 0.678, 0.795), and the sensitivity and specificity were 0.792 and 0.60, respectively (Figs. 3 and 4).

## Discussion

In the recent past, there has been a rapid increase in the number of people suffering from T2D. Thus, research on the occurrence and risk of LEAD in patients with T2D is needed. The symptoms of LEAD associated with T2D are not consistent, with 10% of patients having typical intermittent claudication symptoms, 50% showing symptoms in their lower limbs, and the remaining 40% having no symptoms at all^8^. Therefore, there are few guidelines recommended by the American Heart Association (AHA) or the American College of Cardiology (AC-CF). Accordingly, ABI (ankle-brachial index) screening has been proposed for adults aged 65 years and older who reported having diabetes, non-healing wounds, and a history of smoking. Further, the Diabetes Society of the Chinese Medical Association recommends diabetic patients with risk factors for LEAD should be screened at least once a year. If patients show an ABI >1.3, then calcification of the arterial wall that weakens the ABI is suspected. Therefore, simple ABI examination fails to determine whether the lower extremity arteries have severe stenosis or occlusion. This pseudo-normalized ABI result may lead to a missed diagnosis of LEAD. However, the “gold standard” for the diagnosis of LEAD is digital subtraction angiography (DSA). This requires an invasive examination and injection of contrast agents. Due to the risks involved and cost factors, this method is not suitable for screening high-risk patients^8^. Therefore, we intended to construct a simple-to-use nomogram for visually determining the risk of LEAD in patients with T2D. This tool will aid the detection of high-risk groups at an early stage and thereby enable administration of effective preventive or therapeutic measures to prevent further dysfunction.

Most of the risk assessment methods for LEAD in patients with T2D involve an analysis of risk factors. Mohammedi *et al*. studied 10624 T2D patients and reported that gender, smoking, BP, and lipid metabolism disorder are major risk factors for LEAD^5^. Another study reported that the risk factors for LEAD in patients with T2D are similar to the common risk factors for atherosclerotic disease. This includes traditional and non-traditional risk factors, namely age, smoking, lipid metabolic disorders, hypertension, higher waist-to-hip ratio (WHR), greater carotid intima thickness, and higher levels of CRP, homocysteine, fibrinogen, lipoprotein-A, and D-dimer^6^. Also, diabetes-specific risk factors such as diabetes duration, peripheral neuropathy, blood glucose, and insulin therapy are associated with the development of LEAD^7^. From these studies, multiple risk prediction models have been designed for evaluating the risk of LEAD in patients with T2D. However, due to the limitations of these studies, only a very small number of models are used in clinical practice. The limitations include individualized differences in the population, which lead to a lack of simple and intuitive tools to promote the use of these models. In our study, we have developed nomograms for the first time to describe the risk factors for LEAD in Chinese adult patients with T2D. The nomogram is a complex mathematical formula that has been widely used as a common tool for predicting the prognosis of tumors and cardiovascular diseases^9,10^. Indeed, a few studies have been conducted recently using a nomogram to establish a risk prediction model for the development of T2D in patients with gestational diabetes^11^. However, only a few studies have focused on the prediction of LEAD in T2D patients. The nomograms help to meet our needs for visual tools and personalized prevention. Compared to traditional predictive models, nomograms have a visual digital interface, higher accuracy, and easier-to-understand risk predictions that can be more directly applied to clinical decision-making. Nomograms are simple, fast, inexpensive, and non-invasive, and can be used to effectively identify the risk of developing LEAD in patients with T2D. Upon diagnosis, medical interventions, lifestyle changes, diagnostic management, and treatment can begin. Based on the calculation results, LEAD and moderate-to-severe LEAD predictive models can be established that are convenient to use in clinical practice. Also, we have verified that the two risk assessment models have a certain degree of accuracy internally.

The nomogram developed in this study can directly predict as well as intuitively analyze factors that greatly influence the risk of LEAD in patients with T2D. Diabetic nephropathy and retinopathy are major risk factors among all others for moderate-to-severe LEAD. The risk factors for LEAD and moderate-to-severe LEAD identified in our study are consistent with those found in previous studies. In recent years, age has become considered an uncontrollable risk factor for the development of many chronic diseases. From a study including patients from seven communities in the USA, Allison *et al*. reported that LEAD is rare in men over 50 years, but that its incidence is as high as 20% in men over 80 years. These findings indicate that age is the primary risk factor increasing the incidence of LEAD^12^. Our study used multivariate logistic regression analysis and found that age is an independent risk factor for LEAD in patients with T2D, consistent with the above studies. The possible mechanism is: with increasing age the physiological and endocrine functions of the body are gradually diminished, causing LEAD; in addition, the blood vessels of older adults tend to undergo physiological and structural changes that decrease the utilization rate of nitric oxide (NO) and result in the increased production of angiotensin^8^. However, our study found no statistically significant association between age and moderate-to-severe LEAD.

Various studies on LEAD concluded that the prevalence of this disease in men is usually higher than that among women of the same age group. The reason for this phenomenon may be related to the protective effect of estrogen and cardiovascular risk factors between the different genders^13^. A possible mechanism is: the overall population of diabetes and hypertension patients is relatively old, leading to the gradual increase in the protective effect of the body’s estrogen. In addition, many women reach the menopause age and become prone to other endocrine disorders, leading to an increase in morbidity^14^.

A study of the prevalence of LEAD in 6625 patients with T2D over 50 years of age in five Asian countries, including South Korea, China, Indonesia, Thailand, and Philippines, found that the patients with LEAD had a longer duration of diabetes (p < 0.05), compared to diabetic patients without LEAD^15^. In this study, multivariate logistic regression analysis showed that the duration of T2D remained an independent risk factor for LEAD, which is consistent with the above findings. However, no statistically significant association between moderate-to-severe LEAD and disease duration was observed.

Another prospective study of 5,102 patients with T2D in the United Kingdom found that disease duration and hyperglycemia were associated with the risk of LEAD. In that study, the risk of LEAD increased by 28% (95% CI: 1.12–1.46, p < 0.01) for every 1% increase in the HbA1c level^16^. A meta-analysis followed to confirm the close relationship between hyperglycemia and LEAD in patients with T2D^17^. A prospective study conducted by Althouse *et al*. in patients with T2D recently showed that the hazard ratio (HR) for HbA1c and LEAD was 1.21 (95% CI: 1.12–1.29, p < 0.01)^8^, which was calculated using the Cox proportional hazard model. Univariate logistic regression analysis showed a statistically significant correlation between moderate-to-severe LEAD and HbA1c levels in patients with T2D. Further higher HbA1c level continued to be associated with LEAD and moderate-to-severe LEAD in patients with T2D after adjustment for other factors. The reason may be poor glycemic control over the long-term, which leads to an increase in HbAlc, resulting in the glycation of proteins and thus increasing the formation of advanced glycation end products (AGES). AGES promote the process of arteriosclerosis by oxidative stress of the inflammatory reaction and promote coagulation in other ways, which leads to the occurrence of LEAD in diabetes patients^7^.

Smoking is the primary risk factor for LEAD development, and this has been confirmed in multiple studies^12,18^. We found that smoking was an independent risk factor for LEAD in T2D patients. However, there was no association between moderate-to-severe LEAD and smoking. The main mechanism by which smoking contributes to LEAD may be related to free radical-mediated oxidative stress, which causes atherosclerosis.

Diabetic nephropathy is the most common microvascular complication in diabetic patients, and abnormal urinary albumin is the primary sign of diabetic nephropathy. It is reported that systemic endothelial dysfunction is a common underlying factor for diabetic nephropathy and LEAD^19^. The risk of LEAD in diabetic nephropathy patients is 1.50 times greater than that in non-diabetic nephropathy patients. However, for patients with LEAD, the risk of moderate-to-severe LEAD did not increase. Therefore, active detection of urinary microalbumin is recommended for patients with T2D. If the levels of urinary microalbumin are found to be abnormal, then it is necessary to regularly check and diagnose the lower extremity arteries as proactive care that will enable early treatment for the prevention of LEAD. In our study, the risk factors for moderate-to-severe LEAD were identified based on patients with already diagnosed LEAD. Therefore, it is considered that for this population, risk factors including the level of blood sugar control and the presence of diabetic nephropathy or diabetic retinopathy have more influence on the occurrence of moderate-to-severe LEAD than do age, gender and duration.

Several studies have shown a strong association between retinopathy and macrovascular diseases, such as cardiovascular disease, in diabetic patients^20,21^. On multivariate logistic regression analysis, after adjustment for various factors, showed that retinopathy is an independent risk factor for LEAD and moderate-to-severe LEAD in patients with T2D. This may be related to common risk factors for macrovascular and microvascular diseases. To date, no clear mechanisms have been described for the association between atherosclerosis and retinopathy; however, a few studies have shown that the pathogenesis of these two diseases shares common risk factors^22^.

Although the developed nomograms are a rapid and cost-effective method, this study still has some shortcomings. First, all the patients included in the study were older than 50 years, and young patients with a recent diagnosis were not included. Secondly, this cross-sectional study had difficulty obtaining the sequence of LEAD, and several other research factors could not be determined. Therefore, we recommend that prospective studies are needed to find the relevant causal relationships.

## Methods

### Study design

#### Ethics statement

This study was approved by the Ethics Committee of Peking University International Hospital. All procedures performed in studies involving human participants were in accordance with the ethics standards of the institutional and national research committee and with the 1964 Helsinki Declaration and its later amendments or comparable ethics standards. Written informed consent was obtained from all individual participants included in this study.

#### Study population

This cross-sectional study consecutively enrolled 10681 adults with T2D aged greater than 50 years from 30 hospitals representing different geographic regions of mainland China between June 2016 and January 2017. Among them, 4422 adults had undergone ultrasonography of lower extremities, including 2084 male patients and 2338 female patients.

#### Inclusion and exclusion criteria

The inclusion criteria for participants were: (1) T2DM diagnosis based on the World Health Organization (WHO) guidelines (1999), and (2) age 18–70 years. The exclusion criteria included: (1) type 1, gestational and other types of diabetes; (2) malignant tumors; (3) consciousness disorders, patients with mental problems; (4) heart failure (NYHA class III-IV); (5) previous diagnosis of LEAD; and (6) any disease that the researchers considered would interfere with participation in the study or assessment.

The baseline characteristics of the patients, gender, age, disease duration, height, weight, waist/hip circumference, BMI, T2D history among first-degree relatives, smoking, drinking, coronary heart disease (CHD), and cerebrovascular disease (CBD) as well as the complications of diabetes were recorded in detail. BMI was calculated using the formula BMI (kg/m^2^) = height (kg)/body weight^2^ (m^2^).

### Biochemical examination

Each patient was asked to fast for >8 hours overnight, and a fasting venous blood sample was taken on an empty stomach the next morning. This blood sample was used to test the fasting blood glucose (FBG), total cholesterol (TC), hemoglobin (HbA1c), low-density lipoprotein cholesterol (LDL-C), triglyceride (TG), high-density lipoprotein cholesterol (HDL-C), serum creatinine (SCr), uric acid (UA), and blood urea nitrogen (BUN) levels as well as the estimated glomerular filtration rate (eGFR) based on the SCr level.

### LEAD diagnosis

We used lower extremity arterial color Doppler ultrasound as a diagnostic method for LEAD, with an examination of the femoral, radial, anterior tibia, radial, and dorsal arteries. Scores were calculated based on the condition of the arteries and the extent of stenotic artery lesions. The criteria used were: (1) intima thickening: 0, 1, and 2 points, respectively, for non-thickened (<1 mm), mild thickening (1–1.2 mm) and moderate-to-severe thickening (> 1.2 mm); (2) degree of hardening: 0, 1, and 2 points for normal, mild (no thick endometrium but strong echo, no plaque), and moderate-to-severe sclerosis (mild with plaque or stenosis), respectively; (3) presence of plaque: 0, 1, 2, and 3 points for no plaque, single, multiple, and diffuse plaques, respectively; and (4) stenosis: 0, 1, 2, and 3 points for normal, mild (30–50% stenosis), moderate-to-severe (50–75% stenosis), and occlusion (no blood flow), respectively.

The scores were added to the atherosclerosis score (Grouse score). Based on the total scores, the patients were divided into three groups designated normal (0 points), mild (1–10 points), and moderate-to-severe (11–20 points).

### Statistical methods

Data analyses were performed using SPSS software (version 21.0; SPSS Inc., Chicago, IL, USA). The study data were tested for normal distribution, and data with normal and non-normal distributions were expressed as mean ± standard deviation (SD) and median (interquartile range), respectively. The data were compared among the three patient groups by one-way analysis of variance and Wilcoxon test for normally and non-normally distributed data, respectively. Count data were compared as ratios statistically, and the χ^2^ test was used for comparison among the three groups. Unconditional multivariate logistic regression analysis was used to calculate odds ratios (ORs) and corresponding 95% confidence intervals (95%CIs). All statistical tests were two-sided, and differences were considered statistically significant when p < 0.05.

Receiver operating characteristic (ROC) curves were plotted, and the area under the ROC curve (AUC) values were calculated. To evaluate the discriminatory ability of the nomogram, we computed the AUC with a 95% CI by using 500 bootstrap resamplings. Decision curve analysis was performed to determine the clinical usefulness of the model. Meanwhile, sensitivity and specificity indexes were used to evaluate the significance of nomogram model. All statistical analyses were performed with statistical package R (http://www.R-project.org) and EmpowerStats (www.empowerstats.com, X&Y Solutions, Inc., Boston, MA).

## Data Availability

The datasets generated during the current study are available from the corresponding author on reasonable request.
